# A lesson not learned: allele misassignment

**DOI:** 10.1186/1744-9081-3-65

**Published:** 2007-12-21

**Authors:** Philipp G Sand

**Affiliations:** 1 Department of Psychiatry, University of Regensburg, Germany

## Abstract

Misassigned alleles can annihilate efforts to control quality in otherwise well-designed genetic association analyses. To date, the issue remains underreported, as is exemplified by studies of a diallelic *DRD2 *missense variant in schizophrenia. For this variant, allele frequency data have been either misassigned, or incorrectly cited on four consecutive occasions. Contrary to conjecture, low heterozygosity has not guarded against the error with regard to rs1801028, a SNP that features a canonical base pair transversion, G:C. Measures are discussed that may help to identify misassigned alleles, and to avoid related perils pending more systematic investigation of this confounder in genotype-phenotype associations.

## Background

Conflicting results in case-control association analyses are ascribed to many different factors, including significant phenotypic heterogeneity, population stratification artefacts, inadequate sample size, poorly matched control subjects, polygenic modes of inheritance, epigenetic factors, and multiple testing artefacts, to name only a few [1]. A recent investigation by Vijayan et al. [2] provides the opportunity to highlight inadvertent marker allele misassignment as another source of biased attributable risk estimates. Correct assignment of wildtype and variant allele frequencies is rarely questioned, and has never been the object of systematic study. Therefore, the role played by human errors in reporting data from SNP-based association analyses is essentially unknown despite the public availability of the human genome sequence, and of many reference SNP frequencies to which new data may be compared [3]. In everyday practice, time constraints limit rigorous verification of SNP frequency data during peer review. When results refer to more than a small number of markers, few reviewers can afford to look up reference frequencies. Yet with the advent of haplotypic analyses, the scientific literature has experienced a surge in genetic association studies involving multiple diallelic markers and, at times, hundreds of SNPs [4]. Customized bioinformatic tools for the verification of batch SNP frequency data against the respective reference frequencies in specific ethnic groups are not currently available, and few reviewers are in a position to parse NCBI or other database data according to their needs. Chances are that presently, core data of genetic association studies involving more than one or two markers may pass peer review essentially unscreened for correct allele assignment.

Vijayan et al. [2] have examined *DRD2 *alleles in schizophrenia, using the diallelic Taq1B (rs1079597), Taq1D (rs1800498), S311C (rs1801028), H313H (rs6275), and Taq1A (rs1800497) polymorphisms. To this avail, they have performed PCR-based restriction fragment length polymorphism assays, and have designed PCR primer pairs from alternating DNA strands. Specifically, rs1079597 and rs1800498 were genotyped from the cis strand, whereas the remaining three *DRD2 *variants were assessed in trans. Partly as a result hereof, I presume, wildtype and variant alleles at rs1801028 have been confounded, to judge by HapMap and ALFRED reference data [5,6], plus earlier studies [7,8] in more than 20 populations from around the world. Allele counts at rs1801028 are in disagreement with defining Cys311 as the minor allele (f_Cys311 _= 0.89 and 0.90, for cases and controls, respectively). All 2-, 3-, 4- and 5-marker haplotypes inferred from rs1801028, therefore, are controversial.

## Discussion

A brief review of earlier investigations addressing *DRD2 *SNP data relevant to schizophrenia confirmed that the recent allele misassignment is not an isolated occurrence, and has gone unnoticed for rs1801028 on at least one previous occasion [9]. The repeated misassignment is noteworthy in that it has occurred despite extremely low heterozygosity across most populations (.05, as shown in [6]), which would appear to guard against confounding the wildtype and variant alleles. Rs1801028 encodes for a *DRD2 *missense variant, and alternating allele notations both in IUPAC nucleotide and one-letter amino acid codes may have caused the confusion. Thus the Cys311 variant is sometimes referred to as C311, but is encoded by the nucleotide G, not C, which encodes Ser311. More importantly, the two alleles at rs1801028 are also canonical base pairs, i.e., the variant base is indistinguishable from the wildtype base unless the cis/trans strands have been identified.

Once published, incorrect allele frequencies are easily overlooked. For the rs1801028 G and C allele frequencies, two consecutive reports have inverted the original frequencies without further comment in what may have been an attempt to salvage data for meta-analysis [7,8]. The above papers [2,7-9] thus illustrate the perils of allele assignment, plus the perpetuation of related errors.

What can be done to avoid similar confounders beyond alerting the readership? An important rule of thumb is to minimize data conversion tasks in association studies from the beginning. Authors may be tempted to use surrogate allele identifiers (see [2,9]) that simplify listings of results for multiple variants, or that denote a SNP's functionality at the price of introducing a further data conversion step. Identifiers in IUPAC amino acid one-letter notation should be abandoned in favor of the cis strand base, to be used either alone or in combination with a non-ambiguous surrogate identifier. Finally, notations of variables exclusively in binary code are discouraged, to avoid a format not naturally readable by humans.

If we assume that canonical substitutions are associated with a higher rate of allele misassignment than are non-canonical substitutions, regional differences in substitution patterns may impact on the accurate reporting of genotyping results. Recent estimates of such differences in the human genome have identified regional GC-content as a predictor of substitution rates, primarily with regard to G:C-based transversions [10]. This observation could imply that SNPs from genes with high overall GC-content are more liable to be misassigned when compared to SNPs from genes with only average or low GC-content. However, additional factors will determine whether a given SNP is chosen for genotyping, including its population frequency and its proposed functionality, which may mask such effects.

## Conclusion

Tomorrow's challenges in scientific communication call for a significant upscaling of error-control in data handling, e.g. for genome-scale association studies. With increasingly complex genetic risk interaction models, results from many downstream analyses are at stake when misassigned alleles snowball. Standards are, therefore, a priority. Journal editors may wish to ensure accurate allele assignment in manuscripts to be published by facilatating the task of reviewers. This can be achieved by requesting listings of reference allele frequencies from authors for all SNPs investigated in at least one independent population using earlier reports, or by requesting that genotype data be entered in an online form for the automated retrieval of relevant content from SNP databases. Further quantitative investigation of nonrandomness in allele misassignment is necessary to shed light on the magnitude of confounding effects in association analyses, and to explore more strategies for avoiding such misassignments.

## Competing interests

The author(s) declare that they have no competing interests.
